# Alpha-mangostin inhibits the migration and invasion of A549 lung cancer cells

**DOI:** 10.7717/peerj.5027

**Published:** 2018-06-25

**Authors:** Thi Kieu Trang Phan, Fahimeh Shahbazzadeh, Thi Thu Huong Pham, Takanori Kihara

**Affiliations:** 1 Department of Life and Environment Engineering, Faculty of Environmental Engineering, The University of Kitakyushu, Kitakyushu, Fukuoka, Japan; 2 The Key Laboratory of Enzyme & Protein Technology (KLEPT), VNU University of Science, Vietnam National University, Hanoi, Vietnam

**Keywords:** Co-culture, Cancer invasion, α-mangostin

## Abstract

Several studies have indicated that *α*-mangostin exerts anti-metastasis and anti-subsistence effects on several types of cancer cells. Especially, the anti-metastatic effect of *α*-mangostin on cancer cells is a prospective function in cancer treatment. However, the metastasis process is complicated, and includes migration, invasion, intravasation, and extravasation; thus, the main target of anti-metastatic effect of *α*-mangostin is not known. In this study, we investigated the effects of *α*-mangostin on the invasion, subsistence, and migration of lung cancer cells under co-culture conditions with normal cells and regular mono-culture conditions. We found that *α*-mangostin killed the lung cancer and normal cells in a dose-dependent manner. Furthermore, the alteration in the surface mechanical properties of cells was examined by using atomic force microscopy. Although the *α*-mangostin concentrations of 5 and 10 µM did not affect the short-term cell viability, they considerably decreased the Young’s modulus of lung cancer cells implying a decline in cell surface actin cytoskeletal properties. Additionally, these concentrations of *α*-mangostin inhibited the migration of lung cancer cells. In co-culture conditions (cancer cells with normal cells), the invasive activities of cancer cells on normal cells were discernibly observed, and was inhibited after treatment with 5 and 10 µM of *α*-mangostin. Taken together, *α*-mangostin suppressed the subsistence of lung cancer cells and displayed anti-metastatic activities by inhibiting the migration and invasion, and reducing the actin cytoskeleton of cancer cells. Our findings suggest that *α*-mangostin could be a potential therapeutic agent for cancer treatment.

## Introduction

α-Mangostin is the major xanthone extracted from the pericarp of mangosteen (*Garcinia mangostana Linn*) fruit. Mangosteen is a prevalent fruit in the tropical rainforests of Southeast Asian nations, and its pericarp has had a long history of medicinal value in this region (Pedraza-Chaverri, 2008). The dried pericarp powder has been used as a medicinal agent for treatment of skin-related diseases, wounds, and amoebic dysentery (Chopra, Nayar & Chopra, 1965; Garnett & Sturton, 1932; Mahabusarakam, Wlriyachitra & Taylor, 1987). The pericarp of mangosteen fruit contains a variety of secondary metabolites such as prenylated and oxygenated xanthones (Govindachari et al., 1971; Peres, Nagem & De Oliveira, 2000; Sultanbawa, 1980). Exudates from the mangosteen pericarp include α-, β, and γ-mangostin, garcinone B and E, along with mangostinone, tannins, and a flavonoid called epicatechin (Wexler, 2007). Especially, α-mangostin is the most abundant prenylated xanthone present in the pericarp.

α-Mangostin displays strong pharmacological effects (Ibrahim et al., 2016); specifically, its potential in cancer treatment has attracted increasing attention from scientists. Several studies have indicated that α-mangostin is effective against various types of cancer. α-Mangostin has been shown to induce apoptosis in rat pheochromocytoma (Sato et al., 2004) and human head and neck squamous carcinoma cells (Kaomongkolgit, Chaisomboon & Pavasant, 2011). The anti-proliferative effects of α-mangostin were discovered in human colon cancer (Matsumoto et al., 2005) and canine osteosarcoma cells (Krajarng et al., 2012). The anti-metastatic properties of α-mangostin were found in human prostate carcinoma (Hung et al., 2009), breast adenocarcinoma (Lee et al., 2010), and lung adenocarcinoma cells (Shih et al., 2010). Studies in *in vivo* experiments revealed that α-mangostin reduced the tumor growth and lymph node metastasis (Aisha et al., 2012; Shibata et al., 2011). Thus, α-mangostin is considered to be able to prevent cancer cell metastasis as well as subsistence.

Although a broad range of biological and pharmacological activities of α-mangostin have been reported, the mechanism behind its anti-metastatic effects is not fully understood. In the metastasis process, the cancer cells undergo multiple steps including migration, invasion, intravasation, as well as extravasation (Sahai, 2007). These steps are probable targets for the inhibition of metastasis, especially invasion, which is an early and important target for the inhibition of metastatic process. In this study, we focused on the invasion process of cancer cells and examined the effects of α-mangostin on the progression of initial invasion of cancer cells that come in contact with normal cells. In order to reflect the anti-invasion activities of α-mangostin more accurately in cancer treatment, we established a co-culture system of cancer and normal cells that imitated the initial invasive progression of cancer cells. Lung cancer is one of the most aggressive cancers with a five-year overall survival rate in 10–15% of the patients. This is attributable to the early metastatic process of lung cancer cells via the rapid spread to many distant sites within the body. Therefore, in this study, we employed non-small cell lung cancer A549 cells along with one normal bronchus diploid cell line CCD-14Br and used them in co-culturing experiments.

## Materials and methods

### Materials

Human lung adenocarcinoma cell line A549 cells and normal human bronchus diploid cell line CCD-14Br cells were purchased from Japanese Collection of Research Bioresources (JCRB) cell bank (Osaka, Japan). 3,3′-Dioctadecyloxacarbocyanine perchlorate (DiO), 1,1′-Dioctadecyl-3,3,3′,3′-tetramethylindocarbocyanine perchlorate (DiI), and antibiotics were purchased from Sigma-Aldrich (St. Louis, MO). Cell harvesting solution TrypLE express and fetal bovine serum (FBS) were purchased from Life Technologies Japan Ltd. (Tokyo, Japan). α-Mangostin was purchased from Wako Pure Chemical Industries Ltd. (Osaka, Japan). Cell counting kit-8 was purchased from Dojindo Molecular Technologies, Inc. (Kumamoto, Japan). The cone probe (BL-AC-40TS-C2; spring constant: around 0.05 N/m) was purchased from Olympus (Tokyo, Japan). Other reagents were purchased from Sigma-Aldrich, Wako Pure Chemical Industries Ltd., or Life Technologies Japan Ltd.

### Cell culture

The cells were cultured in DMEM containing 10% FBS and antibiotics (100 units/mL penicillin G and 100 µg/mL streptomycin sulfate) in humidified atmosphere of 95% air and 5% CO_2_ at 37°C

### Determination of cell viability

The viability of cells after treatment with various concentrations of α-mangostin was evaluated by the cell counting kit-8 as recommended by the manufacturer. Briefly, cells were seeded on a 96-well-plate at 10^4^ cells/well (24 h culture experiments) or 1.5 ×10^3^ cells/well (time course experiments) with 100 µL medium and cultured for 24 h, so as to allow the cells to adhere to the plate. The culture medium was replaced by 100 µL of fresh culture medium diluted with various concentrations of α-mangostin for 24–96 h treatment. The medium was replaced by adding 100 µL fresh medium diluted with 10 µL of cell counting kit-8 solution to each well. The cells were cultured for suitable time periods for each cell type. The plate absorbance was then measured at 450 nm using a microplate reader. Ratio of cell viability *V*_*c*_ was calculated as:

\begin{eqnarray*}{V}_{c}= \frac{Ab{s}_{target}-Ab{s}_{background}}{Ab{s}_{ctrl}-Ab{s}_{background}} \end{eqnarray*}

where *V*_*c*_ is the cell viability ratio, *Abs*_target_ is the absorbance of α-mangostin-treated cells, *Abs*_*ctrl*_ is the absorbance of control cells, *Abs*_background_ is the absorbance of the background.

### Measurement of mechanical properties of cells

The cultured cells treated with α-mangostin for 24 h were manipulated by atomic force microscopy (AFM) (Nanowizard III; JPK Instruments AG, Berlin, Germany) at room temperature. Combining optical microscopy (IX-71; Olympus) and AFM allows the probe to be placed on a particular region of the cell surface. In this study, the AFM probe was indented at the top of the cell surface with a loading force of up to 0.5 nN and velocity of 5 µm/s. The Young’s modulus of the cell was calculated using the Hertz model (Hertz, 1881). The force-distance curve for a region up to about 1 µm of cell surface indentation was fitted using JPK data processing software (JPK Instruments AG) as:

\begin{eqnarray*}F= \frac{E}{1-{\nu }^{2}} \frac{2\mathrm{tan}\mathrm{\alpha }}{\pi } {\delta }^{2}, \end{eqnarray*}

Where *F* = force, *δ* = depth of the probe indentation, *ν* = Poisson’s ratio (0.5), α = half-angle of the cone probe (9°), and *E* = Young’s modulus. More than 25 cells were used per experiment, and 25 points were examined on the surface of each cell. The logarithmic Young’s modulus values for each group were compared by nonparametric analyses of variance followed by Kruskal-Wallis *H* test and Steel pairwise comparison test. Young’s modulus of the polystyrene tissue culture surface was more than 1 ×10^7^ Pa (Haghparast, Kihara & Miyake, 2015). The range of Young’s moduli of cell surface was in the order of about 10^2^ to 10^4^ Pa. Thus, we were convinced that the surface stiffness of the cells could be measured by this method without affecting the rigidity of culture surface.

### Wound healing assay

A549 cells were plated on 35-mm culture dishes at a density of 2 ×10^5^ cells using a regular cell culture medium and cultured for 24 h. After the cells achieved confluence, a wound was created by scratching through the middle of the dish with a 200-µL tip (Yuan, Wu & Lu, 2013). Cells were gently rinsed twice with the culture medium to remove any floating cell debris. The medium alone was added to the control dish; the medium diluted with α-mangostin at final concentrations of 5 and 10 µM was added to the treatment dish. The first image acquisition (*t* = 0 h) was then done by using phase contrast microscopy. Cells were then cultured for the next image acquisition (*t* = 12 h and *t* = 24 h). The data were analyzed by ImageJ software (NIH, Bethesda, MD); the ratio of recovered area, *A*_*r*_, which was covered by cells was calculated as

\begin{eqnarray*}{A}_{r}= \frac{{A}_{\mathrm{covered}}}{{A}_{0}} , \end{eqnarray*}

where, *A*_0_ is the scratched area at *t* = 0 h, *A*_covered_ is the area covered cells at different incubation times (*t* = 12, 24 h). The covered areas were compared by nonparametric analyses of variance followed by Kruskal-Wallis *H* test and Steel pairwise comparison test.

### Invasion assay

In order to evaluate the invasion ability of cancer cells, a co-culture system was established by culturing both cancer and normal cell lines together. Briefly, cells were trypsinized, spun, and resuspended with fresh medium. Then, the suspended cells were fluorescently labeled with DiI for A549 cells or DiO for CCD-14Br cells for their membrane at 37°C for 1 h in the dark. Cells were then spun at 200× g for 5 min; the medium was then removed, and cells were resuspended with fresh medium and spun one more time. The labeled cells were cultured in 12-well-plates at a density of 2 ×10^4^ and 4 ×10^4^ cells/well with CCD-14Br and A549 cells, respectively. For monoculture, A549 or CCD-14Br cells were prepared separately at the same density. After incubation for 24 h, the cells were exposed to α-mangostin at 0, 5, and 10 µM concentrations. The cells were observed and image acquisition was done by using fluorescence microscopy at the first time point (*t* = day 0). Then, the cells were cultured for 1, 2, 3, 4, 5, and 6 days and image acquisition was done for each day.

The fluorescence images were analyzed by ImageJ software. After binarization of each fluorescent color image, the cell area estimated by DiI or DiO fluorescence was calculated. Finally, we evaluated the cell area as cell coverage ratio, *A*_*c*_:

\begin{eqnarray*}{A}_{c}= \frac{\text{Fluorescence detectable pixels}}{\text{Total image pixels}} . \end{eqnarray*}

The inverse values of cell doubling time, ${t}_{d}^{-1}$ (*d* ^−1^), were calculated from proliferation slopes in early stage.

## Results

### Cytotoxicity of α-mangostin on A549 and CCD-14Br cells

**Figure 1:**
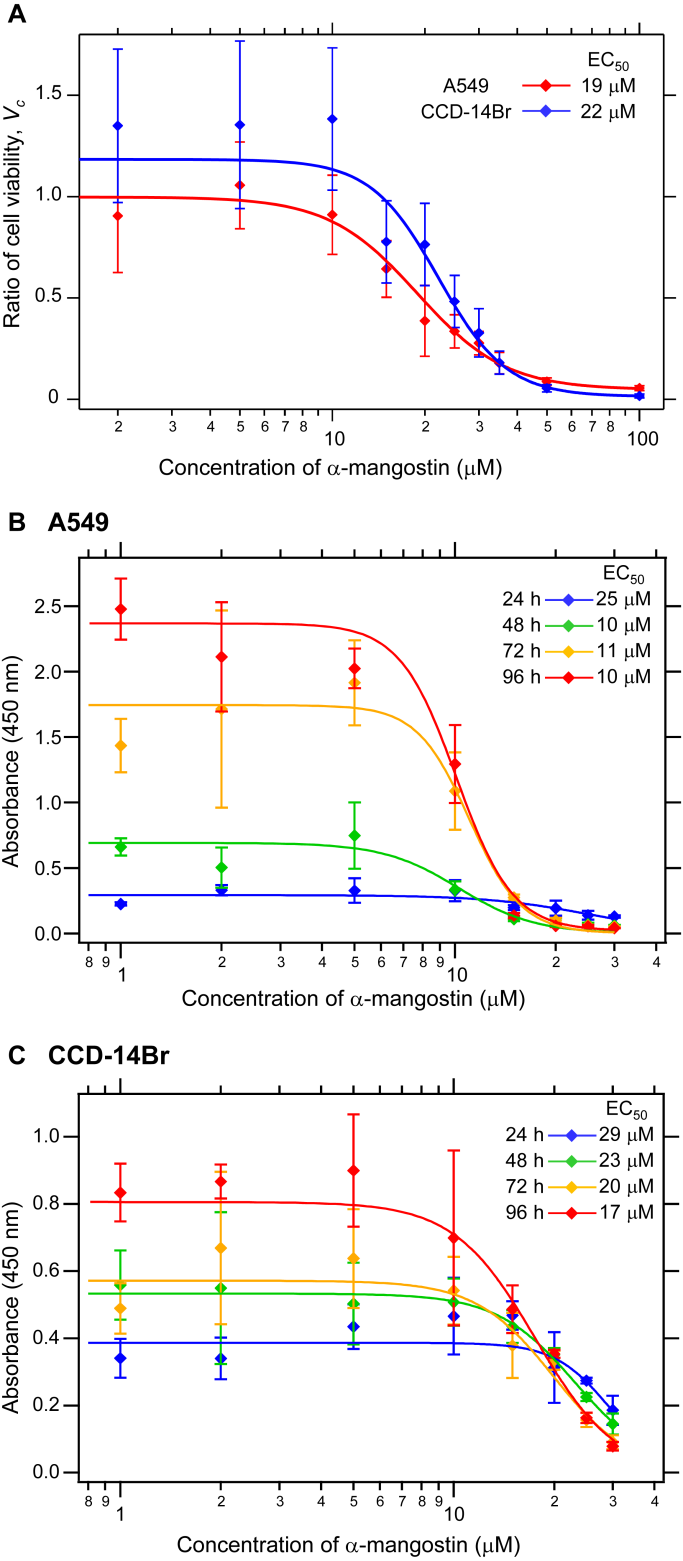
Cytotoxicity of *α*-mangostin on human lung cell lines. (A) Cytotoxicity effects of *α*-mangostin on A549 and CCD-14Br cells in short term period (24 h culture). A549 and CCD-14Br cells were treated with various concentrations of a-mangostin (0–100 µM) incubated for 24 h. The viable cell ratio was measured using the cell counting kit-8. The ratio of cell viability, *V*_*c*_, was expressed as a ratio of *α*-mangostin-treated cells to that of the control. The values were calculated from four experiments. The effective concentration (EC_50_) of A549 and CCD-14Br cells was 19 and 22 µM, respectively. (B) Cytotoxicity effects of *α*-mangostin on A549 cells in time course experiments (24–96 h culture). A549 cells were treated with various concentrations of *α*-mangostin (0–30 µM) incubated for 24–96 h. Viable cell number was evaluated by the cell counting kit-8. The absorbance at 450 nm was plotted in the vertical axis. The values were calculated from four experiments. (C) Cytotoxicity effects of *α*-mangostin on CCD-14Br cells in time-course experiments (24–96 h culture). CCD-14Br cells were treated with various concentrations of *α*-mangostin (0–30 µM) and incubated for 24–96 h. Viable cell number was evaluated by the cell counting kit-8. The absorbance at 450 nm was plotted in the vertical axis. The values were calculated from four experiments.

The cytotoxicity of α-mangostin on A549 cells was evident by a dose- and time-dependent inhibition of cell viability and growth (Shih et al., 2010). We first evaluated the cytotoxicity of α-mangostin by treatment of both the non-small cell lung cancer A549 and pulmonary normal diploid CCD-14Br cell lines with various concentrations of α-mangostin for 24 h. The cell viability was evaluated by the reduction of formazan dye produced from WST-8 in the presence of an electron mediator by the activities of dehydrogenases in cells. α-Mangostin exhibited cytotoxic effects on A549 and CCD-14Br cells at higher concentrations (Fig. 1A). The half-maximal effective concentration (EC_50_) values of α-mangostin for the cytotoxicity of A549 and CCD-14Br cells were 19 µM and 22 µM, respectively (Fig. 1). Furthermore, we examined the cytotoxic effects of α-mangostin on these cells in time course experiments (24–96 h culture). The EC_50_ values of α-mangostin for the cytotoxic effects for A549 and CCD-14Br cells decreased in a time-dependent manner (Figs. 1B and 1C). The EC_50_ values of α-mangostin for the cytotoxic effects for A549 cells almost plateaued at 48 h of culturing and was about 10 µM (Fig. 1B). On the other hand, the EC_50_ values of α-mangostin for the cytotoxic effects for CCD-14Br cells gradually decreased (Fig. 1C). Thus, CCD-14Br cells were less sensitive to the cytotoxic effects of α-mangostin than the A549 cells. Compared with the untreated control, A549 and CCD-14Br cells treated with α-mangostin at concentrations below 5 µM were not significantly different, proving that these dosages are non-toxic to these cells. However, those treated with more than 10 µM of α-mangostin displayed some cytotoxicity (Fig. 1). Therefore, we used less than 10 µM of α-mangostin for subsequent experiments.

### α-Mangostin decreases cell surface stiffness

**Figure 2:**
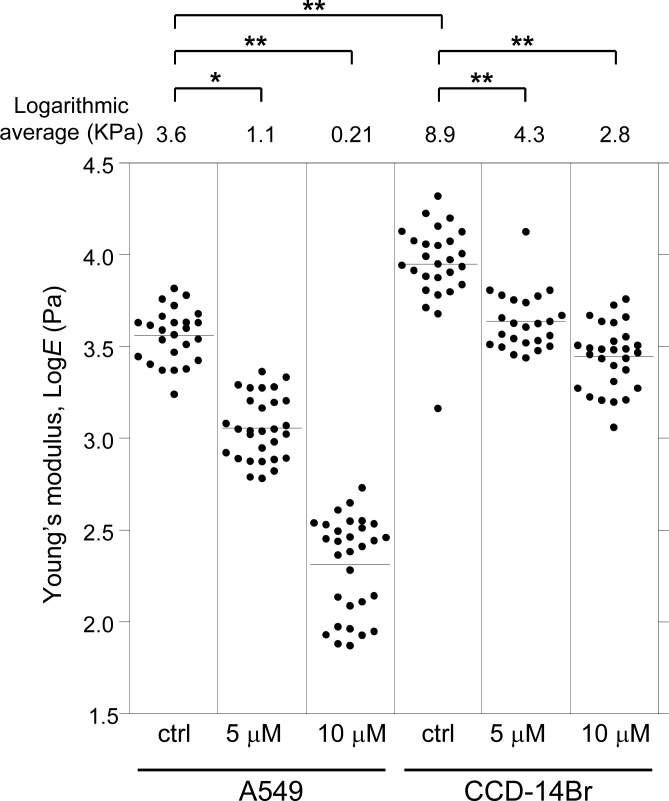
Young’s modulus of A549 and CCD-14Br cells treated with *α*-mangostin. The distribution of the Young’s moduli of cells is shown as scattered plots for treatment with different concentrations of *α*-mangostin for 24 h. The logarithmic average of the Young’s moduli is shown at the top of each plot. Each condition shows the Young’s modulus of more than 25 independent cells, and the surface of each cell was measured 25 times on top of the nucleus. The Kruskal–Wallis *H* value is 138 and *p*-value is less than 0.0001 (*p* < 0.0001). * *p* < 0.05 vs. Young’s modulus of control conditions (Steel pairwise comparison test). ** *p* < 0.01 vs. Young’s modulus of control conditions (Steel pairwise comparison test).

We then examined the effect of α-mangostin on the mechanical properties of cells using AFM. Alterations in cell activities or states often entail a change in the mechanical properties of cells (Haghparast et al., 2013; Shimizu et al., 2012), and the mechanical alterations are largely attributable to the actin cytoskeleton (Dai & Sheetz, 1995; Sugitate et al., 2009). Thus, analyzing the alteration in mechanical properties of cells can reveal the changes in characteristics of their underlying actin networks as well as their states (Haghparast, Kihara & Miyake, 2015; Haghparast et al., 2013; Kihara et al., 2011). AFM indentation is a sensitive method for analyzing the surface mechanical properties of cells (Haghparast, Kihara & Miyake, 2015; Haghparast et al., 2013). Fig. 2 shows the distribution of the Young’s modulus of the cells treated with α-mangostin. The distribution of Young’s modulus of normal CCD-14Br cells (fibroblast-like morphology) has a logarithmic average value of 8.9 kPa, which was clearly higher than that of cancerous A549 cells with logarithmic average value of 3.6 kPa (Fig. 2). The difference in mechanical properties of normal and cancer cells is based on the difference in F-actin structures at the apical surface of these cell types (Cross et al., 2007; Haghparast et al., 2013; Lekka et al., 2012).

Addition of 5 µM or 10 µM of α-mangostin to the cells reduced the distribution of the Young’s moduli in A549 as well as CCD-14Br cells in a dose-dependent manner (Fig. 2). Particularly, the Young’s modulus of A549 cells significantly decreased (Fig. 2). Thus, even though its concentration does not affect cell viability in a short-time period, α-mangostin clearly reduced the surface rigidity of cells. Furthermore, A549 cells, whose surface stiffness was originally soft, were more sensitive to the effect of α-mangostin than the CCD-14Br cells.

### α-Mangostin inhibit cell migration

**Figure 3:**
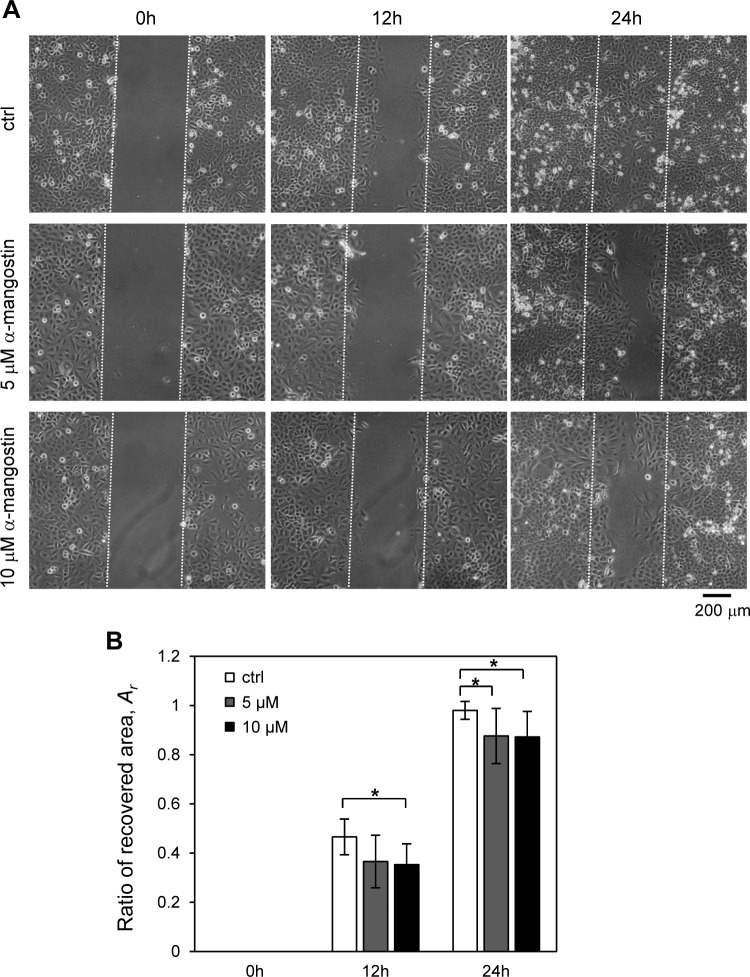
Effect of *α*-mangostin on migration of A549 cells. The cells were treated with 5 and 10 µM *α*-mangostin for 24 h and were subjected to analyses for cell migration. (A) Phase contrast images of wound healing assay of A549 cells treated with *α*-mangostin. The cells were scratched and then cultured for 24 h. The scratched areas are shown with broken lines. (B) The ratio of the recovered wound area, *A*_*r*_, by cell migration. The ratio was calculated from more than 10 images in each condition. *α*-Mangostin displayed an inhibitory effect on cell migration for A549 after 12 and 24 h. The Kruskal-Wallis *H* value and *p*-value of 12 h data are 8.36 and 0.015, respectively. The Kruskal-Wallis *H* value and *p*-value of 24 h data are 8.92 and 0.012, respectively. * *p* < 0.05 vs. *A*_*r*_ of control conditions (Steel pairwise comparison test).

The effect of α-mangostin on lung cancer A549 cell motility was measured by the wound healing assay (Liu et al., 2013), an established method for studying directional cell migration *in vitro*. The migration of several types of cancer cells was reportedly inhibited after treatment with α-mangostin (Shih et al., 2010; Wang, Sanderson & Zhang, 2012; Yuan, Wu & Lu, 2013). In the untreated control group, cells exhibited marked cell migration in the wounded area, whereas the wounds treated with α-mangostin showed a delayed healing (Fig. 3). The ratio of recovered area of wound closure in the untreated cells was about 0.47 at 12 h and almost 0.98 after 24 h (Fig. 3B). On the other hand, α-mangostin, at 5 and 10 µM, reduced the ratio of recovered area of wound closure to approximately 0.36 after 12 h, and 0.87 after 24 h (Fig. 3B). This result indicated that α-mangostin inhibited the migration of A549 cells *in vitro*.

### α-Mangostin suppresses the invasion of cancer cells

**Figure 4:**
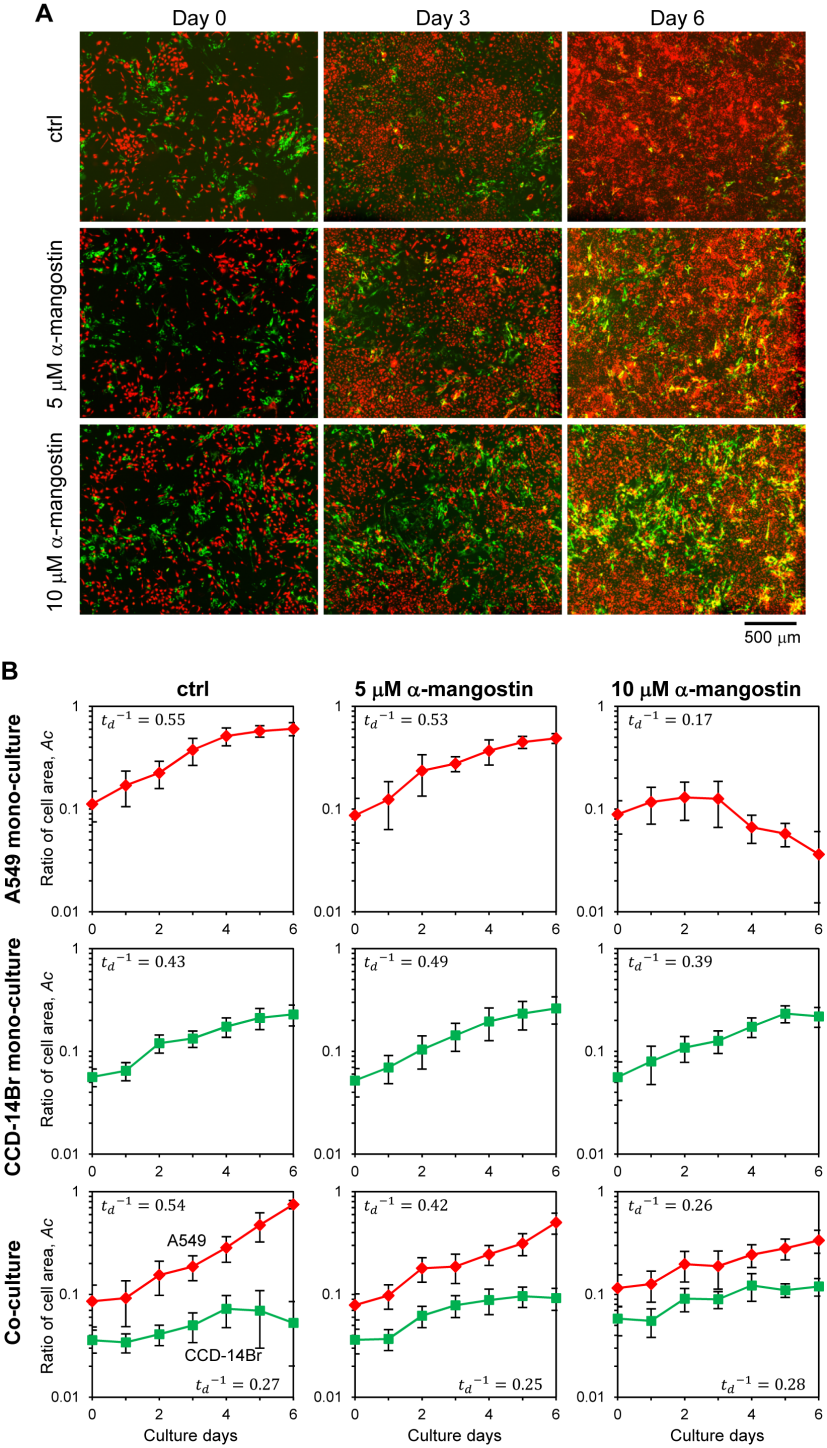
Invasion of cancer A549 to CCD-14Br cells in co-culture conditions treated with *α*-mangostin. Cells were cultured in co-culture or monoculture condition, then exposed with or without *α*-mangostin. (A) Fluorescence images of co-cultured cells. A549 cells were labeled with red fluorescence and CCD-14Br cells were labeled with green fluorescence. (B) Ratio of each cell area, *A*_*c*_, cultured in monoculture or co-culture with A549 and CCD-14Br cells. The cell area was calculated from more than seven fluorescence images in each condition. Inverse value of the doubling time of each culture condition, ${t}_{d}^{-1}$ (*d*^−1^) is shown in each graph.

The co-culturing system of A549 and CCD-14Br cells was used as a model for evaluating cancer cell invasiveness, which imitate the initial invasive progress of cancer cells. Then we examined the potential effects of α-mangostin on cancer cell invasion (Fig. 4A). Besides, the monoculture of each cell type was also conducted to compare their results with those of the co-culture (Fig. S1).

When only cancerous A549 cells were cultured, the cells grew and covered almost all of the plate surface after six days under α-mangostin concentration of less than 5 µM; however, the cells treated with 10 µM of α-mangostin showed a gradual decrease in the area covered by cells (Fig. S1, A549 and Fig. 4B, A549 mono-culture). Conversely, when CCD-14Br cells were cultured under α-mangostin condition, the cells gradually proliferated (Fig. S1, CCD-14Br and Fig. 4B, CCD-14Br mono-culture). These results suggest that A549 cells were relatively sensitive to high concentration of α-mangostin and showed gradual cell death at 10 µM of α-mangostin after three days culture. These results are roughly corresponding to our cytotoxic experiments (Figs. 1B and 1C).

After A549 and CCD-14Br cells were co-cultured, the A549 cells gradually grew and almost covered the whole area at six days (Figs. 4A, ctrl and 4B, Co-culture of ctrl condition). Conversely, the covered area of CCD-14Br cells increased within several days, and after coming in contact with A549 cells, the area of CCD-14Br cells gradually decreased (Figs. 4A, ctrl and  4B, Co-culture of ctrl condition). A549 cancer cells killed and eroded the normal CCD-14Br cells and resulted in a decline in the area of the CCD-14Br cells. In contrast, in the presence of α-mangostin, A549 cells could not cover the whole surface area of the culture plate, and the coverage area of the CCD-14Br cells did not decrease (Figs. 4A, 5 µM of α-mangostin and 10 µM of α-mangostin and 4B, Co-culture of 5 and 10 µM of α-mangostin conditions). Thus, invasive activities of A549 cells on CCD-14Br cells were inhibited by treatment with α-mangostin. Furthermore, in the presence of 10 µM of α-mangostin, A549 cells proliferated gradually but did not show any cell death as seen with the monoculture (Fig. 4B, 10 µM of α-mangostin). Thus, normal CCD-14Br cells probably rescue A549 cells from the cytotoxic effects of highly concentrated α- mangostin. These results demonstrate that by using a co-culturing system, we can conspicuously observe the invasive activities of cancer cells acting on normal cells and α-mangostin exhibited its potential effect in repressing cancer cell invasion.

## Discussion

Metastasis is a critical biological process in cancer pathophysiology and is a therapeutic target for treating active cancer. Previous studies have shown that *α*-mangostin displays anti-metastatic properties in many carcinoma cells and lymph node metastasis (Hung et al., 2009; Lee et al., 2010; Shibata et al., 2011; Shih et al., 2010). Although it has been demonstrated that *α*-mangostin decreased the expression of many cancer-related signal transductions and matrix metalloproteases (Fang et al., 2016; Hung et al., 2009; Krajarng et al., 2011; Shih et al., 2010), the detailed anti-metastatic mechanism of *α*-mangostin remains unclear. One of the reasons is that the metastatic process consists of multiple biological steps including migration, invasion, intravasation, and extravasation; thus, it is difficult to determine which steps are the targets of *α*-mangostin. Previous studies have been conducted only in a monoculture condition, where normal and cancer cells were cultured in isolation. Cancer development, progression, and invasion are positively and negatively affected from the tumor microenvironment, in which cancer cells interact with associated stroma (Quail & Joyce, 2013). However, the monoculture condition alone cannot reproduce the cell responses associated with the cell–cell interactions. In this study, we established a 2D co-culturing system using cancer and normal cells; this model was devised to explore the interactions during the process of lung cancer invasion. Indeed, previous studies demonstrated that the co-culturing system showed dramatically different cellular properties in terms of morphology, proliferation, and cellular function (Angelucci et al., 2012; Furukawa et al., 2015). Thus, the co-culturing system better simulates the living environment and cellular interactions that occur under in vivo conditions. However, our co-culturing system is based on a 2D culture system and thus we are unable to ascertain cancer cell-extracellular matrix interactions. If we want to simulate the whole invasion process of cancer cells, we may have to develop a 3D co-culture system using cancer cells and normal cells in the future. In this study, we demonstrated that *α*-mangostin exerts pharmacological effects in lung cancer treatment, by using not only the monoculture but also co-culture conditions with non-small cell lung cancer A549 cells and normal bronchus diploid CCD-14Br cells.

Our results indicated that A549 cells, which are highly invasive carcinoma cells, invaded and caused serious damage to normal CCD-14Br cells. However, the invasive and erosive activities of A549 cells declined following treatment with *α*-mangostin, which could rescue the CCD-14Br cells from the invaded damage. Another interesting finding was that the A549 cells gradually died when treated for a long time period with high concentration of *α*-mangostin under the monoculture condition, meanwhile the cancer cells survived under co-culture with normal cells. Communication between cancer and surrounding cells is probably mediated by secreted proteins, including growth factors and cytokines (Mueller & Fusenig, 2004; Polyak, Haviv & Campbell, 2009; Quail & Joyce, 2013). Thus, we consider that the surrounding normal cells support the cancer cell subsistence by secreting cytokines. We believe that co-culturing of cancer cells with normal cells provides an environment similar to the tumor microenvironment (Quail & Joyce, 2013) and a more accurate characterization of the invasive ability of cancer cells.

*α*-Mangostin affected cell surface stiffness of A549 and CCD-14Br cells, especially the Young’s modulus of A549 cells, which clearly declined by treatment with 10 µM *α*-mangostin. The surface stiffness of cells reflects their underlying actin networks as well as their states (Haghparast, Kihara & Miyake, 2015; Haghparast et al., 2013; Kihara et al., 2011). Therefore, *α*-mangostin clearly altered the actin network of A549 cancer cells. It is known that the stiffness of cancer cells is lesser than that of the corresponding normal cells (Cross et al., 2008; Guck et al., 2005; Lekka, 2016), and softer cancer cells show higher malignant properties than stiffer cancer cells (Cross et al., 2011; Ramos et al., 2014). On the other hand, F-actin modification reagents usually decrease cell migration (Yamaguchi & Condeelis, 2007), and apoptotic cells are less stiff than normal cells (Kihara et al., 2009; Kim et al., 2012). *α*-Mangostin-treated cancer cells presented a decrease in their migration and invasion properties. Thus, *α*-mangostin probably affects the F-actin structures or mass, and this change has negative effects on cancer cell properties.

Finally, we discuss the pharmacological potential of *α*-mangostin. We suggest that *α*-mangostin shows an ability to suppress cancer cells at concentrations about 10 µM, which could effectively inhibit cancer progression by inhibiting cell growth, migration, and invasion. Using this dosage of *α*-mangostin, we aim to treat cancer by turning off the growth and development of cancer cells. However, only low levels of *α*-mangostin are adsorbed through the gastrointestinal tract in treated mice and the bioavailability *F* value of *α*-mangostin from oral administration is about 0.8% (Choi et al., 2014). The terminal half time of *α*-mangostin after intravenous administration is about 3 h in mice (Choi et al., 2014). For further research and effective application of *α*-mangostin in cancer treatment, it is necessary to develop an efficient system to deliver the optimal amount of *α*-mangostin to the cancer-affected area in our body.

## Conclusions

We demonstrated that *α*-mangostin exerts pharmacological effects in lung cancer treatment, by using monoculture and co-culture conditions with non-small cell lung cancer A549 cells and normal bronchus diploid CCD-14Br cells. The EC_50_ values of *α*-mangostin cytotoxicity on A549 cells were lower than those of CCD-14Br cells. Although the dosages below 10 µM of *α*-mangostin did not show significant toxicity in an early 24-h cell culture, the treatment clearly affected A549 cancer cell properties. *α*-Mangostin decreased surface stiffness and inhibited the migration of A549 cells. Furthermore, *α*-mangostin repressed cancer cell invasion in normal cells. Our findings thus suggest that *α*-mangostin could be a potential therapeutic agent for cancer treatment. Furthermore, we established a co-culturing system using cancer and normal cells, and this model was devised to explore the interactions involving the cancer cell and normal cell as cancer invades.

##  Supplemental Information

10.7717/peerj.5027/supp-1 Figure S1 Fluorescence images of normal monoculture of A549 and CCD-14Br cells A549 cells were labeled with red fluorescence and CCD-14Br cells were labeled with green fluorescence.

10.7717/peerj.5027/supp-2 Data S1 Raw data

## Additional Information and Declarations

### Data Availability

The following information was supplied regarding data availability: The raw data is provided in a Supplemental File.
