# Supplementary figures and images for: Alpha-mangostin inhibits the migration and invasion of A549 lung cancer cells
Source: PeerJ. 2018 Jun 25;6:e5027. doi: 10.7717/peerj.5027 (PMC6022730; doi:10.7717/peerj.5027)

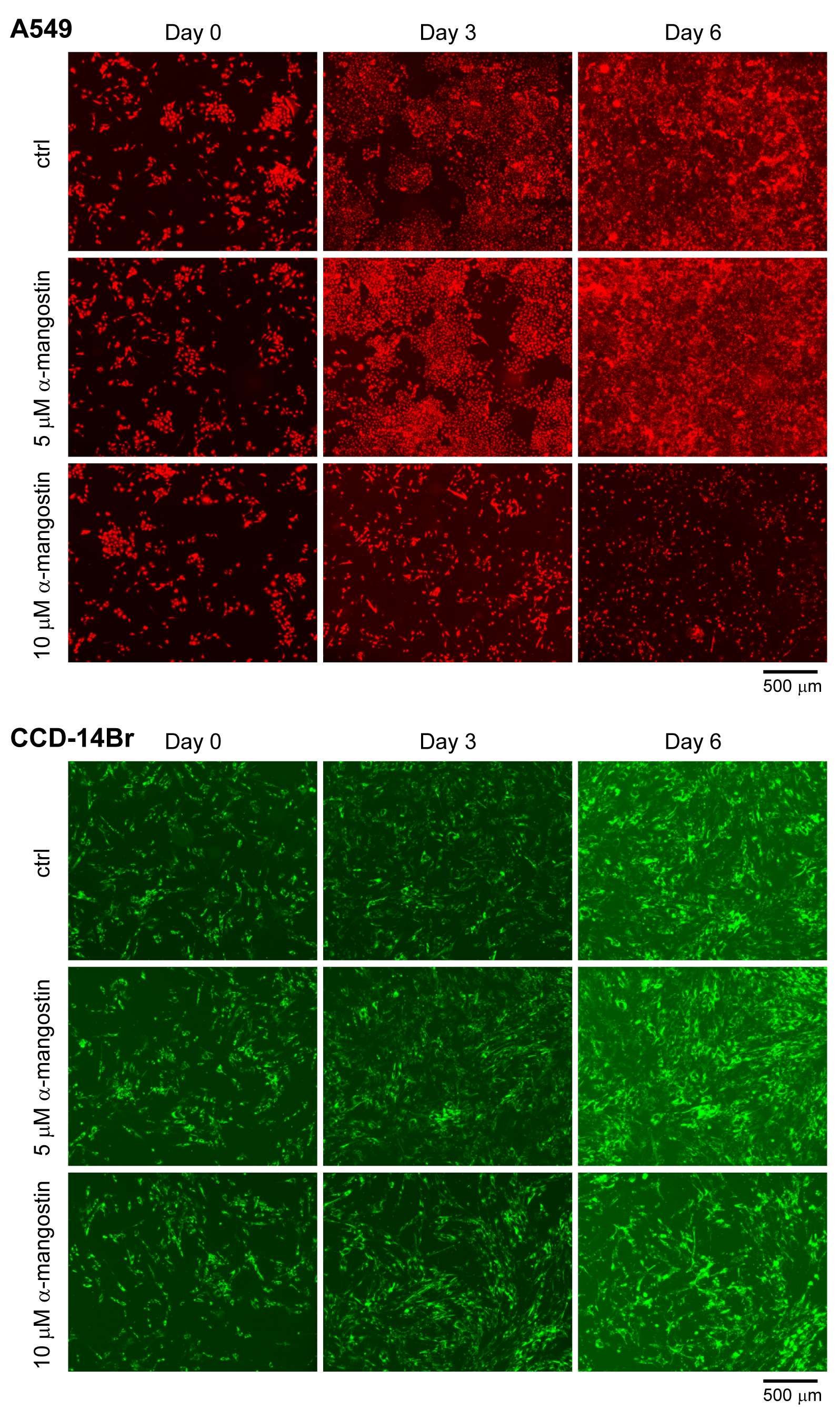

Supplement: Figure S1 — A549 cells were labeled with red fluorescence and CCD-14Br cells were labeled with green fluorescence. [file peerj-06-5027-s001.png]
